# Lessons from past pandemics: a systematic review of evidence-based, cost-effective interventions to suppress COVID-19

**DOI:** 10.1186/s13643-022-01958-9

**Published:** 2022-05-12

**Authors:** Carl-Etienne Juneau, Tomas Pueyo, Matt Bell, Genevieve Gee, Pablo Collazzo, Louise Potvin

**Affiliations:** 1 Direction Régionale de Santé Publique, CIUSSS du Centre-Sud-de-l’Île-de-Montréal, Montréal, QC Canada; 2 COVID-19 Work Group, Washington, D.C., USA; 3 Danube University, Dr. Karl Dorrek Straße 30, 3500 Krems, Austria; 4 École de Santé Publique, Université de Montréal, C.P. 6128, Succursale Centre-ville, Montréal, QC H3C 3J7 Canada

**Keywords:** Non-pharmaceutical interventions, Epidemic interventions, Outbreak control, Pandemic control, Cost-effectiveness, COVID-19

## Abstract

**Background:**

In an unparalleled global response, during the COVID-19 pandemic, 90 countries asked 3.9 billion people to stay home. Yet other countries avoided lockdowns and focused on other strategies, like contact tracing. How effective and cost-effective are these strategies? We aimed to provide a comprehensive summary of the evidence on past pandemic controls, with a focus on cost-effectiveness.

**Methods:**

Following PRISMA guidelines, MEDLINE (1946 to April week 2, 2020) and EMBASE (1974 to April 17, 2020) were searched using a range of terms related to pandemic control. Articles reporting on the effectiveness or cost-effectiveness of at least one intervention were included.

**Results:**

We found 1653 papers; 62 were included. The effectiveness of hand-washing and face masks was supported by randomized trials. These measures were highly cost-effective. For other interventions, only observational and modelling studies were found. They suggested that (1) the most cost-effective interventions are swift contact tracing and case isolation, surveillance networks, protective equipment for healthcare workers, and early vaccination (when available); (2) home quarantines and stockpiling antivirals are less cost-effective; (3) social distancing measures like workplace and school closures are effective but costly, making them the least cost-effective options; (4) combinations are more cost-effective than single interventions; and (5) interventions are more cost-effective when adopted early. For 2009 H1N1 influenza, contact tracing was estimated to be 4363 times more cost-effective than school closure ($2260 vs. $9,860,000 per death prevented).

**Conclusions and contributions:**

For COVID-19, a cautious interpretation suggests that (1) workplace and school closures are effective but costly, especially when adopted late, and (2) scaling up as early as possible a combination of interventions that includes hand-washing, face masks, ample protective equipment for healthcare workers, and swift contact tracing and case isolation is likely to be the most cost-effective strategy.

**Supplementary Information:**

The online version contains supplementary material available at 10.1186/s13643-022-01958-9.

## Key points

- Randomized trial evidence was only available to support the effectiveness of hand-washing and face masks, both highly cost-effective measures. For other interventions, only evidence from observational and modelling studies was available.
- This lower-quality evidence suggests that overall, when timed appropriately, the following interventions are likely to be highly cost-effective: contact tracing and case isolation, protective equipment for healthcare workers, and vaccination prior to the outbreak (when available). Surveillance networks and protective equipment for healthcare workers also appear to be cost-effective.
- The least cost-effective interventions appear to be social distancing measures, like workplace and school closures. The evidence suggests that these are more cost-effective for severe viruses and when timed early in the outbreak.

## Background

On March 11, 2020, the World Health Organization (WHO) characterized COVID-19 as a pandemic. In an unparalleled global response, more than 90 countries or territories have asked about half of the world’s population to stay home [1]. During that time, over 1.5 billion (almost 90%) of the world’s students were affected by nationwide school closure [2]. Some countries focused on other interventions, such as contact tracing, which has been estimated to be 4363 times more cost-effective than school closure for H1N1 influenza ($2260 vs. $9,860,000 per death prevented) [3]. Indeed, closing school is costly—$10 to $47 billion for 4 weeks in the US alone [4]. As countries around the world are faced with the ongoing challenge of balancing public health interventions with economic, ethical, social, and legal considerations, evidence on the effectiveness and cost-effectiveness of these interventions is needed to guide policy and avoid unnecessary harm.

An earlier systematic review of non-pharmaceutical interventions to reduce influenza transmission in adults included only randomized trials, analysed 7 studies, and concluded that the evidence was lacking for most interventions [5]. While we do not dispute this conclusion when looking only at randomized trials, we would argue that as decisions of unknown cost-effectiveness are made in reaction to the COVID-19 pandemic, some evidence, even if of lower quality, is better than no evidence at all. Therefore, we turned to past pandemics and included a broad range of study designs in this review to provide a comprehensive summary of the evidence on epidemic control, with a focus on cost-effectiveness, to draw lessons applicable to COVID-19.

## Methods

Following PRISMA systematic review guidelines [6], MEDLINE (1946 to April week 2, 2020) and EMBASE (1974 to April 17, 2020) were searched using the terms “non-pharmaceutical interventions”, “outbreak control”, “outbreak interventions”, “epidemic control”, “epidemic interventions”, “pandemic control”, and “pandemic interventions” (last search: April 19, 2020). Reference lists and PubMed-related articles of included studies were reviewed to find additional articles. Reviews (all types), randomized trials, observational studies, and modelling studies were included. Articles reporting on the effectiveness or cost-effectiveness of at least one intervention were included. We defined effectiveness as success in producing the desired outcome, and cost-effectiveness as doing so with minimum economic cost (in dollar value). Articles in English, French, Spanish, and Portuguese were included. Studies of sexually transmitted infections (e.g. syphilis) and mosquito-borne diseases (e.g. dengue) were not included. Abstracts, case reports, and conference proceedings were also excluded. Hand-washing and face masks were the focus of a number of reviews [5, 7, 8] and a recent meta-analysis [9, 10], so individual studies of their effectiveness were also excluded. Likewise for school closure [11–14]. Titles were screened by a single investigator. Abstracts and full texts were screened by two investigators. Discrepancies were solved by mutual agreement. Key characteristics of studies were recorded in a spreadsheet, including first author, year of publication, study design, interventions studied, and a summary of findings. Quality assessment was limited to grouping studies based on design into two categories: higher quality (randomized trials) and lower quality (other designs). Meta-analysis was not feasible due to the heterogeneous set of interventions studied, as well as substantial differences in study designs, outcomes, and effect measures. We synthesized results narratively.

## Findings

### Result of the systematic review

A total of 2742 papers were found. Removing duplicates left 1653. We retained 622 based on title, 137 based on abstract, and 39 based on full text. We found 23 additional studies via reference lists and PubMed-related article searches (eFigure in the Supplement). Therefore, a total of 62 studies were included (Table 1). Randomized trial evidence was only available to support the effectiveness of hand-washing and face masks [5, 7–10]. For other interventions, only lower-quality (observational and modelling) evidence was available.

**Table 1:** Study characteristics

Last name of the first author	Year of publication	Study design	Intervention(s)	Key finding(s)
Ahmed	2018	Review	Workplace social distancing	Modelling studies estimated workplace social distancing reduced the cumulative influenza attack rate 23%.
Armbruster	2007	Modelling	Contact tracing Screening	Contact tracing is cost-effective only when population prevalence is low (e.g. under 8% for tuberculosis)
Barnes	2007	Modelling	Case isolation Personal protection equipment Antiviral prophylaxis	Antiviral prophylaxis can contain an influenza strain with R0 = 2; healthcare workers contribute disproportionately to the transmission of the infection when not protected against infection.
Becker	2005	Modelling	Face masks Social distancing Hand hygiene Case isolation School closures Quarantining affected households Contact tracing	Combinations more effective than single interventions; household quarantines and contact tracing reduced reproduction number from 6 to below 1.
Bell	2006	Review	Travel restrictions	Screening and quarantining entering travellers were ineffective in past pandemics; WHO recommends providing information to international travellers and screening travellers departing infected countries.
Bell	2006	Review	School closures Isolation of patients Quarantine of contacts Antiviral therapy Travel restrictions Hand hygiene Respiratory hygiene Face masks Disinfected household surfaces	At the start of the outbreak, detect and isolate cases, quarantine contacts, restrict travel in and out of affected communities, and consider targeted antiviral therapy. If sustained transmission in the general population, ill persons are advised to remain at home; increase social distance; promote hand-washing and respiratory hygiene/cough etiquette; wear face masks; and disinfect contaminated household surfaces.
Bin Nafisah	2018	Review	School closures	School closures reduced the peak of epidemics by an average of 29.65%.
Bolton	2012	Modelling	School closures Travel restrictions Generalized social distancing Quarantining of close contacts Treatment of cases with antivirals Prophylaxis of contacts	A combination of non-pharmaceutical interventions proved as effective as the targeted use of antivirals and reduced the mean attack rate from approximately 23 to 21% (severe pandemic scenario).
Bootsma	2007	Observational	School closures Church closures Mass gatherings Face masks Case isolation Disinfection/hygiene measures	The timing of interventions during the 1918–1919 flu pandemic (specifically early intervention) was the most strongly correlated factor with total mortality. Population size and density were not significant factors in overall mortality.
Buonomo	2012	Modelling	Health promotion campaign Vaccination	Early vaccination is more cost-effective; interventions before vaccines are available need to be balanced with the potential gains in the cost-effectiveness of future vaccines.
Caley	2007	Modelling	Border screening Face masks during transit Immediate presentation following symptom onset Flight-based quarantining	The most effective strategy to prevent spread is control in the source country; targeting travellers is not effective.
Chen	2020	Modelling	School closure Case isolation Antiviral treatment Antiviral prophylaxis Vaccination	Case isolation was the most effective single intervention, adding antiviral therapeutics, antiviral prophylaxis, vaccination prior to the outbreak, and school closure decreased the attack rate only slightly and shortened outbreak duration by only 9 days
Chinazzi	2020	Modelling	Travel restrictions	90% travel restrictions to and from China only modestly affect the epidemic trajectory unless combined with a 50% or higher reduction of transmission in the community.
Chong	2012	Modelling	Travel restrictions (air, land, sea) Antiviral treatment	Restricting air travel from infected regions 99% delays epidemic peak up to 2 weeks. Restricting air and land travel delays peak ~3.5 weeks. Neither 90% nor 99% travel restrictions reduced the epidemic magnitude more than 10%. Antiviral treatment and hospitalization of infectious subjects are more effective.
Dan	2009	Modelling	Personal protection equipment Personal protection equipment plus restricting visitors and cancelling elective procedures	Personal protection equipment cost-effective for H1N1 ($23,600 per death prevented) Personal protection equipment plus restricting visitors and cancelling elective procedures less cost-effective ($2500,000 per death prevented)
Day	2006	Modelling	Quarantine	Quarantine of all contacts is beneficial only when case isolation is ineffective, significant asymptomatic transmission, and asymptomatic period is neither very long nor short.
Figueroa	2017	Observational	Mass gatherings	Outbreaks at mass gatherings were uncommon, even during the 2009 H1N1 pandemic
Gostic	2020	Modelling	Traveller screening	Even under best-case assumptions, screening (at border or locally) will miss more than half of people infected with SARS-CoV-2.
Halder	2011	Modelling	School closures Workplace closure Antiviral treatment Household antiviral prophylaxis Extended antiviral prophylaxis	Combinations were more cost-effective than single interventions; best combination included treatment and household prophylaxis using antiviral drugs and limited duration school closure ($632 to $777 per case prevented)
Halton	2013	Review	Surveillance Contact tracing Isolation and quarantine	Contact tracing and progressively earlier isolation of probable SARS cases were associated with control of the SARS outbreak.
Handel	2006	Modelling	Hypothetical interventions	Interventions before vaccines are available must be balanced with potential gains of future vaccines or potential multiple outbreaks.
Hellewell	2020	Modelling	Contact tracing	Highly effective contact tracing and case isolation enough to control a COVID-19 outbreak within 3 months. Transmissibility is an important factor (when Ro = 2.5, 80%+ contacts needed to be traced and isolated). Timing is also important (5 initial cases, 50%+ chance of achieving control, even at modest contact-tracing levels; however, 40 initial cases, control much less likely). Delays from symptom onset to isolation decreased the probability of control.
Herrera-Diestra	2019	Modelling	Vaccination	Vaccinating (or self isolating) based on the number of infected acquaintances is expected to prevent the most infections while requiring the fewest intervention resources.
Ishola	2011	Review	Mass gatherings	Some evidence to indicate that mass gatherings may be associated with an increased risk of influenza transmission
Jackson	2014	Review	School closures	School closures are most effective when they cause large reductions in contact, when the basic reproduction number is below 2, and when attack rates are higher in children than in adults
Jefferson	2011	Review	Screening at entry ports Isolation Quarantine Social distancing Barriers Personal protection Hand hygiene	Overall masks were the best performing intervention across populations, settings, and threats
Lee	2009	Review	Antivirals Antibiotics Vaccination Case isolation Quarantine Personal hygiene measures Social distancing Travel restrictions	Combinations delayed spread, reduced the overall number of cases, and delayed and reduced peak attack rate more than individual strategies.
Lee	2011	Modelling	Hand hygiene Disinfectant measures Patient isolation Personal protection equipment Staff exclusion policies Ward closures	Implementing increased hand hygiene, using protective apparel, staff exclusion policies, or increased disinfection separately or in bundles provided net cost-savings, even when the intervention was only 10% effective in preventing further norovirus transmission. Patient isolation or ward closure was cost-saving only when transmission prevention efficacy was very high (≥90%), and their economic value decreased as the number of beds per room and the number of empty beds per ward increased.
Lee	2010	Modelling	Vaccination	Vaccination priority should be given to at-risk individuals and to children within high-risk groups
Li	2013	Modelling	Quarantine of close contacts	Quarantine in Beijing during 2009 H1N1 reduced confirmed cases by a factor of 5.6; given that H1N1 was mild, “not economically effective”.
Lin	2010	Modelling	Social distancing Case isolation	Supports early containment; the best strategy depends on the transmission characteristics of virus, the state of the pandemic, and the cost and implementation levels of intervention.
MacIntyre	2019	Modelling	Case isolation Contact tracing	Outbreak controlled in 100 days when 95% of cases isolated and 50% of contacts traced.
MacIntyre	2015	Review	Face masks	Face masks provide protection against infection in various community settings
Markel	2007	Observational	School closures Isolation or quarantine Public gathering ban	Cities that implemented interventions earlier had greater delays in reaching peak mortality (Spearman *r*=−0.74, *P*<0.001), lower peak mortality rates (Spearman *r*=0.31, *P*=.02), and lower total mortality (Spearman *r*=0.37, *P*=.008). A significant association between increased duration of interventions and a reduced total mortality burden (Spearman *r*=−0.39, *P*=.005).
Martinez	2014	Modelling	School closures Workplace closures Case isolation Household quarantine	School closure was the single most effective intervention; combination of all interventions was most effective.
Mateus	2014	Review	Travel restrictions	Evidence did not support travel restrictions as an isolated intervention for the containment of influenza.
Nguyen	2018	Modelling	Vaccination	Vaccination should be administered 5 months before to 1 week after the start of an epidemic.
Pan	2020	Observational	Traffic restrictions Cancellation of social gatherings Home quarantines Designated hospitals and wards Personal protective equipment Increased testing capacity Quarantine of presumptive cases Quarantine of confirmed cases and of their close contacts	Traffic restrictions, cancellation of social gatherings, and home quarantines are associated with reduced transmission, but not sufficient to prevent increases in confirmed cases. Ro reduced below 1 only when all interventions are implemented.
Pasquini-Descomps	2017	Review	School closures Disease surveillance networks Contact tracing and case isolation Face masks Preventive measures in hospitals Antiviral treatment Antiviral prophylaxis Vaccination Stockpiling antiviral medicine quarantining confirmed cases at home Self-isolation at home	The most cost-effective interventions were disease surveillance networks and contact tracing and case isolation; the least cost-effective intervention was school closure.
Perlroth	2010	Modelling	School closures Quarantine of infected individuals Child social distancing Adult social distancing Antiviral treatment Antiviral prophylaxis	Combinations were more cost-effective than single interventions; the best combination included adult and child social distancing, school closure, and antiviral treatment and prophylaxis ($2700 per case).
Prosser	2011	Modelling	Vaccination	Incremental cost-effectiveness ratios ranged from $8000 to $52,000 per quality-adjusted life year for persons aged 6 months to 64 years without high-risk conditions
Rainey	2016	Review	Mass gatherings	Mass gathering-related respiratory disease outbreaks were relatively rare between 2005 and 2014 in the US, suggesting low transmission at most types of gatherings, even during pandemics
Rashid	2015	Review	School closures	School closures moderately effective in reducing influenza transmission and delaying epidemic peak; associated with very high costs
Ryu	2020	Review	Travel restrictions	Evidence does not support entry screening as an effective measure.
Sang	2012	Modelling	Quarantine Isolation Entry travel screening	Isolation was the best strategy; entry screening delays the peaks but does not prevent the epidemic.
Saunders-Hastings	2017	Modelling	School closure Community-contract reduction Hang hygiene Face mask Voluntary isolation Quarantine Vaccination Antiviral prophylaxis Antiviral treatment	Vaccination plus antiviral treatment most cost-effective (cost per life-year saved: $2581); however, it still led to 3026 life-years lost. Only 1607 life-years lost at a marginally higher cost ($6752) with combination including community-contact reduction, hand hygiene, face masks, voluntary isolation, and antiviral therapy. Combining all interventions saved most lives (267 life-years lost), but very costly ($199,888 per life-year saved).
Saunders-Hastings	2017	Review	Hand hygiene Face masks	Hand hygiene significant protective effect (OR = 0.62; 95% CI 0.52–0.73). Face masks non-significant protective effect (OR = 0.53; 95% CI 0.16–1.71) (randomized control trials and cohort studies) Face masks significant protective effect (OR = 0.41; 95% CI 0.18–0.92) (randomized control trials and cohort studies pooled with case–control studies)
Schiavo	2014	Review	Communicating health risk Promoting disease control measures	Evidence not conclusive
Shi	2010	Modelling	Mass gatherings	Mass gatherings that occur within 10 days before the epidemic peak can result in a 10% relative increase in peak prevalence and total attack rate; little effect when occurring more than 40 days earlier or 20 days after the infection peak (when initial Ro = 1.5)
Shiell	1998	Modelling	Vaccination	Vaccinating all unvaccinated school-aged children was the most cost-effective strategy ($32.90 marginal cost per case prevented).
Smith	2015	Review	School closure Quarantine Social distancing Oral hygiene Hand hygiene Face masks Social gatherings	Positive significant interventions included professional oral hygiene intervention in the elderly and hand washing.
Suphanchaimat	2020	Modelling	Vaccination	Incremental cost-effectiveness ratio of vaccination (compared with routine outbreak control) $1282–$1990/DALY.
Townsend	2017	Modelling	Hand hygiene	National behaviour change programme would net $5.6 billion (3.4–8.6) in India and $2.64 billion (2.08–5.57) in China
Tracht	2012	Modelling	Face masks	$573 billion saved if 50% of the US population used masks in an unmitigated H1N1 epidemic
Tuncer	2018	Modelling	Isolation Quarantine Education Safe burial Social distancing	Social distancing had the most impact on the 2014 Ebola epidemic in Liberia, followed by isolation and quarantine.
Van Genugten	2003	Modelling	Vaccination Antiviral treatment	Similar results from vaccinating the entire population vs. only at-risk groups; best strategy combined pneumococcal vaccination of at-risk groups and antiviral treatment.
Velasco	2012	Review	School closure Antiviral prophylaxis Social distancing Vaccination Quarantine	Combinations were more cost-effective than vaccines and/or antivirals alone; reducing non-essential contacts, using pharmaceutical prophylaxis, and closing schools was the most cost-effective combination.
Viner	2020	Review	School closures	School closures did not help the control of the 2003 SARS epidemic in China, Hong Kong, and Singapore and would prevent only 2–4% of COVID-19 deaths
Young	2019	Modelling	Isolations and quarantines	Case isolation is likely ineffective when the identification of infected hosts is not sufficiently thorough or delayed.
Zhang	2015	Modelling	Voluntary self-isolation Antivirals	Voluntary self-isolation at symptom onset can achieve the same level of effectiveness as starting antiviral prophylaxis; when delayed 2 days, strategy has a limited effect on reducing transmission.
Zhang	2012	Observational	Border screening Close contact tracing (and quarantine) Medical follow-up of international travellers Influenza-like illness monitoring	Border screening: 132/600,000 (0.02%) people infected; contact tracing: 120/4768 (2.5%) infected; medical follow-up of international travellers: 18/346, 847 (0.005%) infected; influenza-like illness monitoring: 339/180,495 (0.2%) infected.
Zhao	2020	Observational	Domestic travel	Each increase of 100 in daily new cases and daily passengers departing from Wuhan was associated with an increase of 16.25% (95% CI: 14.86–17.66%) in daily new cases outside of Wuhan.

### Cost-effectiveness of interventions

Pasquini-Descomps et al. [15] conducted a systematic review of the cost-effectiveness of interventions in H1N1 influenza. They found 18 studies covering 12 interventions: disease surveillance networks (very cost-effective), contact tracing and case isolation (very cost-effective), face masks (very cost-effective), preventive measures in hospitals (cost-effective), antiviral treatment (cost-effective), antiviral prophylaxis (cost-effective), low efficiency vaccination (cost-effective if timed before cases peak), high efficiency vaccination (cost-effective if timed before cases peak), stockpiling antiviral medicine (cost-effective for high-income countries), quarantining confirmed cases at home (cost-effective for viruses with a case fatality rate of 1%, not cost-effective for viruses with a case fatality rate of 0.25%), self-isolation at home (cost-effective with a case fatality rate of 1%, not cost-effective with a case fatality rate of 0.25%), and school closure (not cost-effective). Based on these findings, Madhav et al. [3] estimated that for H1N1 influenza, contact tracing was 4363 times more cost-effective than school closure ($2260 vs. $9,860,000 per death prevented). Other systematic reviews found that school closures did not help control of the 2003 SARS epidemic in China, Hong Kong, and Singapore and would prevent only 2–4% of COVID-19 deaths [14]; reduced the peak of epidemics by 29.65% on average and were more effective when timed early [11]; are most effective when they cause large reductions in contact, when the basic reproduction number is below 2, and when attack rates are higher in children than in adults [12]; and appeared to be moderately effective in reducing the transmission of influenza and in delaying the peak of an epidemic, but were associated with very high costs [13]. Differences in publication date, virus transmissibility, and study selection may explain the discrepancies among these reviews.

Using data from Wang et al. [16] and Pasquini-Descomps et al. [15] found that contact tracing and case isolation was one of the most cost-effective interventions to control H1N1 in Hubei, China (less than $1000 per disability-adjusted life year). In a simulation study, Hellewell et al. [17] found that in most scenarios, highly effective contact tracing and case isolation would be enough to control a new outbreak of COVID-19 within 3 months. Transmissibility was an important factor, i.e. when Ro = 2.5, 80% of contacts needed to be traced and isolated. Timing was another important factor—with five initial cases, there was a greater than 50% chance of achieving control, even at lower contact-tracing levels. However, at 40 initial cases, control was much less likely. Similarly, any delay from symptom onset to isolation decreased the probability of control, highlighting the need for swift action. Furthermore, Armbruster and Brandeau [18] found that contact tracing is cost-effective only when population prevalence is still low (e.g. under 8% for tuberculosis). In a systematic review, Halton et al. [19] found that contact tracing and progressively earlier isolation of probable SARS cases were associated with the control of SARS outbreaks in Southeast Asia. In another review, Bell et al. [20] recommended contact tracing and case isolation at the start of an outbreak, but not in the late phase, when there is increased and sustained transmission in the general population. In a modelling study, Zhang et al. [21] found that voluntary self-isolation at symptom onset can achieve the same level of effectiveness as antiviral prophylaxis, but that this strategy had a limited effect on reducing transmission when delayed by 2 days. Young et al. [22] also found that delays could prevent case isolation from stopping incipient outbreaks. Li et al. [23] found that in the 2009 H1N1, quarantine of close contacts in Beijing reduced confirmed cases by a factor of 5.6. However, since H1N1 was mild, they concluded that this was not an economically effective measure. In another modelling study, Tuncer et al. [24] found that social distancing had the most impact on the 2014 Ebola epidemic in Liberia, followed by isolation and quarantine. Case isolation, household quarantine, and contact tracing were the most effective interventions in four other modelling studies [25–28]. Collectively, in the context of COVID-19, these studies suggest that these interventions can be effective and cost-effective, and highly so when implemented early and executed swiftly.

Saunders-Hastings et al. [9, 10] carried out a systematic review and meta-analysis of personal protective measures to reduce pandemic influenza transmission. Meta-analyses suggested that regular hand hygiene provided a significant protective effect (OR = 0.62; 95% CI 0.52–0.73). Face masks had a non-significant protective effect (OR = 0.53; 95% CI 0.16–1.71), which became significant (OR = 0.41; 95% CI 0.18–0.92) when randomized control trials and cohort studies were pooled with case–control studies (this also decreased heterogeneity). In an earlier systematic review, Jefferson et al. [7] also found a protective effect of masks. Overall, they were the best performing intervention across populations, settings, and threats. Similarly, in a narrative review, MacIntyre and Chughtai [8] drew on evidence from randomized community trials to conclude that face masks do provide protection against infection in various community settings, subject to compliance and early use. Differences in publication date, search strategy, and study selection criteria may explain the discrepancies among these reviews. Tracht et al. [29] estimated savings of $573 billion if 50% of the US population used masks in an unmitigated H1N1 epidemic. For hand-washing, Townsend et al. [30] estimated that a national behaviour change programme in India would net $5.6 billion (3.4–8.6), a 92-fold return on investment. A similar programme in China would net $2.64 billion (2.08–5.57), a 35-fold return on investment.

Preventive measures in hospitals include the use of personal protective equipment for healthcare workers in direct contact with suspected patients. Dan et al. [31] estimated that this measure was cost-effective for H1N1 ($23,600 per death prevented). However, adopting a wider set of measures (full personal protective equipment, restricting visitors, and cancelling elective procedures) was much less cost-effective ($2,500,000 per death prevented). Similarly, Lee et al. [32] found that increasing hand hygiene, use of protective apparel, and disinfection are the most cost-saving interventions to control a hospital outbreak of norovirus. If they are not adequately protected, healthcare workers can contribute disproportionately to the transmission of the infection [33].

Suphanchaimat et al. [34] found that influenza vaccination for prisoners in Thailand was cost-effective. The incremental cost-effectiveness ratio of vaccination (compared with routine outbreak control) was $1282 to $1990 per disability-adjusted life year. Shiell et al. [35] also found that vaccination (for measles) was cost-effective ($32.90 marginal cost per case prevented). Prosser et al. [36] also found that H1N1 vaccination in the US was cost-effective under many assumptions if initiated prior to the outbreak. Incremental cost-effectiveness ratios ranged from $8000 to $52,000 per quality-adjusted life year for persons aged 6 months to 64 years without high-risk conditions. The authors noted that all doses (two for some children, one for adults) should be delivered before the epidemic peak. Similarly, in a modelling study, Nguyen et al. [37] found that vaccination should be administered 5 months before to 1 week after the start of an epidemic to be cost-effective. If vaccine supplies are limited, Lee et al. [38] found that priority should be given to at-risk individuals and to children within high-risk groups. Likewise, Van Genugten et al. [39] estimated similar results from vaccinating the entire population versus only at-risk groups. Herrera-Diestra and Meyers [40] found that vaccinating based on the number of infected acquaintances is expected to prevent the most infections while requiring the fewest intervention resources. Optimal control modelling studies also suggest that early intervention and vaccination are more cost-effective and that interventions before vaccines are available need to be balanced with the potential gains of future vaccines or the potential for multiple outbreaks [41–43].

In another systematic review of economic evaluations, Pérez Velasco et al. [44] examined 44 studies and found that combinations of pharmaceutical and non-pharmaceutical interventions were more cost-effective than vaccines and/or antivirals alone. Reducing non-essential contacts, using pharmaceutical prophylaxis, and closing schools was the most cost-effective combination for all countries. However, quarantine for household contacts was not cost-effective, even in low- and middle-income countries. A modelling study by Day et al. [45] suggested that quarantine (of all individuals who have had contact with an infected individual) would be beneficial only when case isolation is ineffective, when there is significant asymptomatic transmission, and when the asymptomatic period is neither very long nor very short.

Perlroth et al. [46] estimated the health outcomes and costs of combinations of 4 social distancing strategies and 2 antiviral medication strategies. For a virus with a case fatality rate of 1% and a reproduction number of 2.1 or greater, school closure alone was the least cost-effective intervention and cost $32,100 per case averted. Antiviral treatment ($18,200), quarantine of infected individuals ($15,300), and adult and child social distancing ($5600) had increasing levels of cost-effectiveness. However, combining interventions was more cost-effective, and the most cost-effective combination included adult and child social distancing, school closure, and antiviral treatment and prophylaxis ($2700 per case). However, the same combination without school closure was more cost-effective for milder viruses (case fatality rate below 1%, reproduction number 1.6 or lower). If antivirals are not available, the combination of adult and child social distancing and school closure was most effective. Similarly, in another modelling study, Bolton et al. [47] found that a combination of non-pharmaceutical interventions proved as effective as the targeted use of antivirals.

In a similar study of cost-effectiveness, Saunders-Hastings et al. [10] examined a range of interventions (school closure, community-contract reduction, hand hygiene, face mask, voluntary isolation, quarantine, vaccination, antiviral prophylaxis, antiviral treatment) in response to a simulated pandemic similar to the 1957 H2N2. In a population of 1.2 million, with no intervention, 9421 life-years were lost. Vaccination plus antiviral treatment was the most cost-effective intervention (cost per life-year saved: $2581). However, it still led to 3026 life-years lost. Only 1607 life-years were lost at a marginally higher cost ($6752 per life-year) with a combination of interventions including community-contact reduction, hand hygiene, face masks, voluntary isolation, and antiviral therapy. Combining all interventions saved the most lives (only 267 life-years lost), but was very costly ($199,888 per life-year saved) due to school closure and workdays lost.

Halder et al. [48] aimed to determine the most cost-effective interventions for a pandemic similar to H1N1. They found that a combination of interventions was the most cost-effective. This combination included treatment and household prophylaxis using antiviral drugs and limited duration school closure ($632 to $777 per case prevented). If antiviral drugs are not available, limited duration school closure was significantly more cost-effective compared to continuous school closure. Other social distancing strategies, such as reduced workplace attendance, were found to be costly due to productivity losses. Closing school for 2 to 4 weeks without other interventions did not cost much more than doing nothing but gave a significant 34 to 37% reduction in cases, if optimally timed.

### Studies on intervention effectiveness without cost-effectiveness analysis

Smith et al. [5] carried out a systematic review of non-pharmaceutical interventions to reduce the transmission of influenza in adults. Only randomized trials were included, and 7 studies met all selection criteria. The authors found that positive significant interventions included professional oral hygiene intervention in the elderly and hand-washing, and noted that home quarantine may be useful, but required further assessment.

Jefferson et al. [7] conducted a Cochrane systematic review of physical interventions to interrupt or reduce the spread of respiratory viruses. They found that the highest quality randomized cluster trials suggested this could be achieved by hygienic measures such as hand-washing, especially around younger children. They recommended that the following effective interventions be implemented, preferably in a combined fashion, to reduce transmission of viral respiratory disease: frequent hand-washing with or without adjunct antiseptics; barrier measures such as gloves, gowns, and masks with filtration apparatus; and suspicion diagnosis with isolation of likely cases.

Lee et al. [49] carried out a systematic review of modelling studies quantifying the effectiveness of strategies for pandemic influenza response. They found that combinations of strategies increased the effectiveness of individual strategies and could reduce their potential negative impact. Combinations delayed spread, reduced the overall number of cases, and delayed and reduced peak attack rate more than individual strategies. Similar results were found by Martinez and Das [50]. In another systematic review of 12 modelling and three epidemiological studies, Ahmed et al. [51] found that workplace social distancing reduced cumulative influenza attack rate by 23%. It also delayed and reduced the peak attack rate.

Pan et al. [52] examined associations between public health interventions and the epidemiology of COVID-19 in Wuhan, China. Traffic restrictions, cancellation of social gatherings, and home quarantines were associated with reduced transmission, but were not sufficient to prevent increases in confirmed cases. These were reduced and estimates of the effective reproduction number fell below 1 only when additional interventions were implemented. Those included hospital-based measures (designated hospitals and wards, use of personal protective equipment, increased testing capacity, accelerated reporting, and timely medical treatment) and community-based interventions (quarantine of presumptive cases and quarantine of confirmed cases of their close contacts in designated facilities).

Markel et al. [53] examined non-pharmaceutical interventions in US cities during the 1918–1919 influenza pandemic (isolation or quarantine, school closure, public gathering ban). They found that all 43 cities in the study adopted at least one of these interventions and that 15 cities applied all three. The most common combination (school closure and public gathering bans) was implemented in 34 cities (79%) for a median duration of 4 weeks and was significantly associated with reductions in weekly excess death rate. Cities that implemented interventions earlier had greater delays in reaching peak mortality (Spearman *r*=−0.74, *P*<0.001), lower peak mortality rates (Spearman *r*=0.31, *P*=.02), and lower total mortality (Spearman *r*=0.37, *P*=.008). There was a significant association between increased duration of interventions and a reduced total mortality burden (Spearman *r*=−0.39, *P*=.005). Another similar, historical study of US cities found that early intervention was associated with lower mortality (*R*^2^=0.69, *P*<0.01) [54].

Ishola and Phin [55] reviewed the literature on mass gatherings. They found 24 studies and cautiously concluded that there is some evidence to indicate that mass gatherings may be associated with an increased risk of influenza transmission. In a more recent systematic review, Rainey et al. [56] found that mass gathering-related respiratory disease outbreaks were relatively rare between 2005 and 2014 in the US. They concluded that this could suggest—perhaps surprisingly—low transmission at most types of gatherings, even during pandemics. Similarly, in a US survey of 50 State Health Departments and 31 large local Health Departments, Figueroa et al. [57] found that outbreaks at mass gatherings were uncommon, even during the 2009 H1N1 pandemic. In a modelling study, Shi et al. [58] found that mass gatherings that occur within 10 days before the epidemic peak can result in a 10% relative increase in peak prevalence and total attack rate. Conversely, they found that mass gatherings may have little effect when occurring more than 40 days earlier or 20 days after the infection peak (when initial Ro = 1.5). Thus, the timing of mass gatherings might explain the apparent lack of evidence in support of their ban.

Recently, Zhao et al. [59] quantified the association between domestic travel out of Wuhan, China, and the spread of SARS-CoV-2. Using location-based data, they estimated that each increase of 100 in daily new cases and daily passengers departing from Wuhan was associated with an increase of 16.25% (95% CI: 14.86–17.66%) in daily new cases outside of Wuhan. Ryu et al. [60] conducted a systematic review of international travel restrictions, screening of travellers, and border closure. They examined 15 studies and concluded that the evidence did not support entry screening as an effective measure and that travel restrictions and border closures would have limited effectiveness in controlling pandemic influenza. In another systematic review, Mateu et al. [61] concluded that the evidence did not support travel restrictions as an isolated intervention for the containment of influenza and that restrictions would be extremely limited in containing the emergence of a pandemic virus. Chong and Ying Zee [62] modelled the impact of travel restrictions on the 2009 H1N1 pandemic in Hong Kong. They estimated that restricting air travel from infected regions by 99% would have delayed the epidemic peak by up to 2 weeks. Restricting both air and land travel (from China) delayed the peak by about 3.5 weeks. However, neither 90% nor 99% travel restrictions reduced the epidemic magnitude by more than 10%, and antiviral treatment and hospitalization of infectious subjects were found to be more effective than travel restrictions. Chinazzi et al. [63] modelled the impact of travel limitations on the spread of COVID-19. They estimated that the travel quarantine of Wuhan delayed the overall epidemic progression by 3 to 5 days in mainland China and reduced international case importations by nearly 80% until mid-February. In addition, sustained 90% travel restrictions to and from China only modestly affected the epidemic trajectory, unless combined with a 50% or higher reduction of transmission in the community. Bell et al. [64] point out that screening international travellers who depart infected countries (instead of all travellers entering all countries) would be a better use of resources. Case in point: Zhang et al. [65] reported that in the 2009 H1N1, only 132 of the 600,000 travellers who underwent border entry screening in Beijing were infected (0.02%). Travel limitations may be more effective when neighbouring countries fail to implement adequate outbreak control efforts [66, 67].

We found little evidence to support the following interventions: (1) communicating health risk and promoting disease control measures in low- and middle-income countries (evidence not conclusive according to a review by [68]); (2) screening to contain spread, at the borders or locally (even under best-case assumptions, more than half of infected people would be missed, according to a modelling study by [69]).

## Discussion

This systematic review aimed to provide a comprehensive summary of the evidence on pandemic control, with a focus on cost-effective interventions in the context of COVID-19. Randomized trial evidence was only available to support the effectiveness of hand-washing and face masks, both highly cost-effective measures during past pandemics. For other interventions, only evidence from observational and modelling studies was available. This lower-quality evidence suggests that overall, when timed appropriately, the following interventions were likely to be highly cost-effective: contact tracing and case isolation, protective equipment for healthcare workers, and vaccination prior to the outbreak (when available). Surveillance networks and protective equipment for healthcare workers also appeared to be cost-effective. Home quarantine for confirmed cases and stockpiling antivirals appeared less cost-effective. The least cost-effective interventions appeared to be social distancing measures like workplace and school closures. However, the evidence suggests that these could still be cost-effective when timed early in the outbreak, and when viruses were severe (with high mortality or morbidity, leading to high costs). Vaccination past the peak of infections and long-term school closure late in the outbreak appeared less cost-effective, underscoring the importance of timing.

What lessons can policymakers learn from past pandemics? Three major underlying themes stand out. First, timing and preparedness. Our findings suggest interventions are more effective when timed early. But paradoxically, some interventions may take months, or even years to prepare (e.g. establishing effective disease surveillance networks). This highlights the importance of pandemic preparedness. Learning from past pandemics, policymakers may be well advised to develop ahead of time clear, actionable pandemic response plans and to allocate the necessary human, financial, and logistical resources. Second, individual vs. population-level interventions. In general, interventions that focus on individuals appear more cost-effective (e.g. promoting hand-washing, tracing contacts, and providing personal protective equipment for healthcare workers). In contrast, interventions that apply to entire populations appear less cost-effective (e.g. closing workplaces and schools). Individual-level interventions may also be more feasible and acceptable. Indeed, infected individuals may comply with targeted interventions more readily than entire populations, in which healthy and otherwise well-functioning people may come to question the legitimacy of public health measures, especially when heavy-handed and long-lasting, thus raising ethical and legal considerations. Third, at-risk groups. In general, interventions that target individuals appear even more cost-effective when they focus on at-risk groups (e.g. prioritizing at-risk individuals for vaccination). For COVID-19, at-risk groups include older people and those with chronic diseases. Programmes like the UK’s shielding scheme have focused on these groups [70]. By definition, at-risk groups have the most potential for prevention, and they would also seem more likely to welcome interventions. Still, from a critical perspective, all the above raises important questions. How far are we willing to go to save a life? How much are we willing to spend to do so? How much are we willing to restrict freedom, and for how long? And are all lives equally worth saving? The answer to these questions should be made explicit, and policymakers in democratic countries may wish to consult the population before assuming that all lives should be saved at all cost. Finally, these findings can be further criticized for the strength of the evidence in their support, or lack thereof. Indeed, as higher-quality evidence was only available to support hand-washing and face masks, policymakers would be hard-pressed to justify the continued use of all control measures in any pandemic based on scientific evidence alone. Should policymakers wish to do so, we believe they should communicate transparently about the evidence base, and all the other factors weighing on their decision-making process.

How can these lessons from past pandemics be translated to the current COVID-19 pandemic? Key differences emerge. The incubation period is longer for COVID-19 (6.4 days) than for influenza type A (3.4 days) [71]. This poses challenges, as cases can infect others during the incubation period. Likewise, while only about 20% (95% CI: 17–25) of cases remain asymptomatic throughout infection, asymptomatic transmission does occur, albeit at a lower rate (relative risk: 0.35, 95% CI 0.10–1.27) [72]. This in turn poses substantial challenges to one of the most cost-effective measures in past pandemics, namely contact tracing. Indeed, modelling studies suggest that to stop the spread of COVID-19, public health practitioners only have 2–3 days from the time a new case develops symptoms, to isolate the case and quarantine its contacts [73]. Otherwise, cases tend to surge, and tracing efforts can become overwhelming. This may explain why many countries failed to control COVID-19 with contact tracing—the UK, for example, spent ten billion pounds on its test and trace programme, which may not have been effective [74].

Another key difference between COVID-19 and influenza is the duration of hospitalization. It is longer for COVID-19 (14 days) than for influenza (6.5–6.7 days) [71]. This may partially explain why intensive care units around the world were overburdened during the COVID-19 pandemic. Public health officials therefore aimed to “flatten the curve”. In doing so, when all other control measures fell short, they sometimes used the least cost-effective interventions of past pandemics (workplace and school closures). Past pandemics indicate that these measures are more cost-effective when timed early in the outbreak, and when caseloads are severe. That they have been used at multiple times before and during outbreaks in this pandemic, and for various durations, may explain the wide range of estimates calculated for their cost-effectiveness during the COVID-19 pandemic. These range from net benefits of $5.2 trillion [75] to costs being “at least 5–10 times” greater than benefits ([76], p.1). While their cost-effectiveness is still debated, two studies have found that less disruptive (and economical) interventions can be as effective as more restrictive (and costly) ones [77, 78]. The cost-effectiveness of interventions also depends on virus severity. For SARS-CoV-2, estimates of case fatality rates range from 1 to 7.2% [79], making it more severe than influenza and other respiratory viruses of past pandemics. To some extent, this may justify more costly measures.

Another defining characteristic of the COVID-19 pandemic is the emergence of virus variants, leading to concerns of immunity escape [80]. At the time of writing, the WHO designates four variants of concern: Alpha, Beta, Gamma, and Delta [81]. These evolve under selective pressure, are more transmissible, and may escape immunity conferred by infection or vaccination. Indeed, data suggest that some vaccines are less effective against variants B.1.351 (Beta) [82] and B.1.617.2 (Delta) [83]. Evidence from previous pandemics indicates that vaccinating past the peak of infections may not be cost-effective, yet in the current COVID-19 pandemic, as the ability to develop vaccines more rapidly has become apparent, along with multiple waves of infections, it might still be worth vaccinating past the peak, as there may be future waves once restrictions are eased. While there is no doubt that in most jurisdictions, the majority of the population has not yet been infected, some data suggest that COVID-19 can surge even in areas with high seroprevalence from past infection (e.g. 76% in Manaus, Brazil [84];) or high vaccination (e.g. 78% in Israel [85];). In this context, vaccine cost-effectiveness may be lower than hoped, especially if annual booster shots are needed [86]. Likewise, if other measures fail to prevent surges due to variants, workplace and school closures, if they are to be used again, should be timed early as daily new cases surge.

Cost-effectiveness is also shaped by cultural and behavioural responses to interventions. Culture awareness has arguably become a critical input to the successful design and implementation of effective and equitable health policies [87, 88]. Cultural and behavioural traits, while largely overlooked in the literature, are likely to play a pivotal role in shaping policy responses and assessing sanitary outcomes in the ongoing COVID-19 pandemic. Empirical evidence supports this otherwise intuitive claim, notably Erman and Medeiros’ [89] meta-study of 73 countries, accounting for ca. 93% of confirmed cases and 96% of deaths directly attributed to COVID during the first wave of the pandemic (up to September 2020), as cultural/behavioural attributes (e.g. uncertainty avoidance and long-term vs. short-term normative orientation) significantly impact public health outcomes (i.e. crude test positivity, case/infection fatality, and mortality risk). Along the same lines, a study of 1140 residents of the UK and Ireland, accounting for a culturally diverse sample across the Americas (North, Central and South), Asia and Europe, provides evidence on significant mean differences (MANCOVA) in physical and mental behaviours during the pandemic, attributable to cultural differences [90], arguably reinforcing the claim that cost-effectiveness should be assessed through cultural and behavioural lenses.

**Fig. 1:**
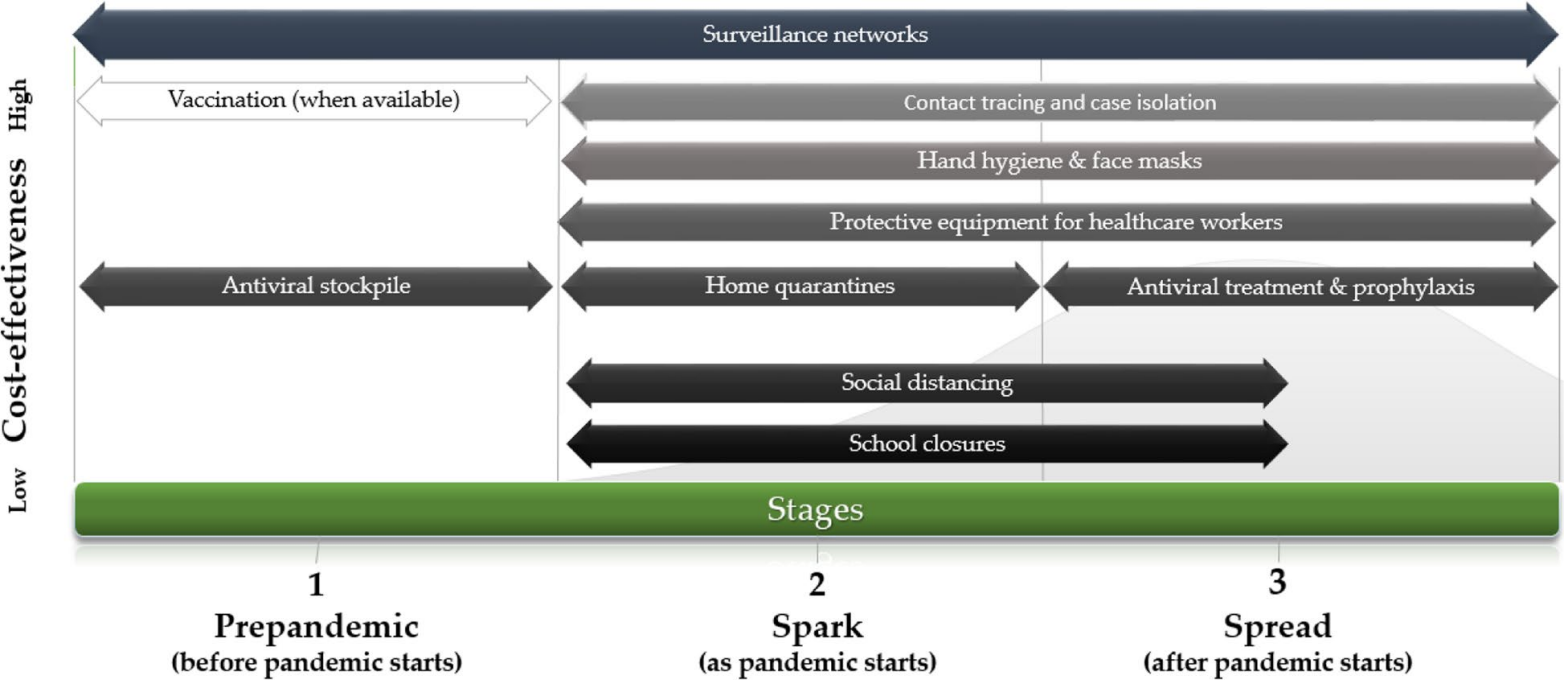
Cost-effectiveness of interventions in COVID-19, by stage

As noted, the cost-effectiveness of interventions depends on their timing and virus severity. Taking this into account, we propose a 3-stage framework for cost-effective control of COVID-19 (Fig. 1). Interventions are shown from top (most cost-effective) to bottom (least cost-effective), according to the three stages described by Madhav et al. [3] as pre-pandemic, spark, and spread (shown from left to right). A complete description is found in the Supplement.

### Strengths and limitations

This review arguably has one key strength: turning to past pandemics, it included a broad range of study designs to provide a comprehensive summary of the evidence. This could also be viewed as a limitation, as the evidence for many measures is of lower quality. Lower-quality evidence should be interpreted with caution. Still, as randomized trial evidence was not available for most pandemics, and as COVID-19 forces urgent decision-making, we submit that some evidence, even if of lower quality, is better than no evidence at all. Moreover, studying these interventions during a pandemic poses substantial methodological challenges, and it may not be possible, physically or ethically, to conduct them under a trial design. In addition, this review has a number of limitations. First, as we deemed it relevant to promptly release our results, our search was limited to two databases (MEDLINE and EMBASE). Second, we did not assess the risk of bias. Third, we studied past pandemics, not COVID-19. Past pandemics have limited generalizability to COVID-19. Fourth, the COVID-19 pandemic is a rapidly evolving situation, and estimates of COVID-19 case fatality rates are subject to substantial uncertainties—especially due to variants. Should the true rate be high, all interventions would be more cost-effective. Conversely, should it be low, costly interventions such as workplace and school closures may not be cost-effective at all. Similarly, estimates of cost-effectiveness based solely on case fatality rates ignore the potential for long-term morbidity, i.e. “long COVID” [91]. To the extent that this phenomenon proves to be widespread, debilitating, and long-lasting, all interventions may become more cost-effective in hindsight. Fifth, interventions studied during past pandemics of a smaller scale may not be readily feasible during the COVID-19 pandemic, given its scale and the relative lack of preparedness of some jurisdictions—as illustrated by shortages of face masks early on in the pandemic and limited contact tracing capabilities.

Among the noteworthy, non-sanitary side-effects of the ongoing COVID-19 pandemic, the need for a broader perspective on the socio-economic costs unveiled by the disease stands out as a call for action to multiple stakeholders, particularly policymakers. Yet most of those costs remain currently hidden, as they relate to unknown morbidities subsequent to the infection, and on an aggregate note, are contingent on the resilience of the social and economic fabric of the given country or region. Hence, in order to provide a more factual assessment of cost-effectiveness, we relied on the disability-adjusted life year (DALY) as a measure of health burden, a metric extensively used in academia and policymaking.

## Conclusions

Hand-washing and face masks were the only measures supported by higher-quality evidence. Other interventions were supported by lower-quality evidence. In the context of COVID-19, a cautious interpretation suggests that (1) workplace and school closures are effective but costly, especially when adopted late, and (2) scaling up as early as possible a combination of interventions that includes hand-washing, face masks, ample protective equipment for healthcare workers, and swift contact tracing and case isolation is likely to be the most cost-effective strategy.

## Supplementary Information

**Additional file 1.** Result of the search. Description of 3-stage framework.

**Additional file 2.** PRISMA Flow Diagram.

## Data Availability

All data generated or analysed during this study are included in this published article [and its supplementary information files].

## Abbreviations

COVID: Coronavirus disease
EMBASE: Excerpta Medica dataBASE
H1N1: Hemagglutinin type 1 and neuraminidase type 1 (influenza strain)
H2N2: Hemagglutinin type 2 and neuraminidase type 2 (influenza strain)
ICU: Intensive care unit
MEDLINE: Medical Literature Analysis and Retrieval System Online
PRISMA: Preferred Reporting Items for Systematic Reviews and Meta-Analyses
SARS: Severe acute respiratory syndrome
SARS-CoV-2: Severe acute respiratory syndrome coronavirus 2
US: United States of America
WHO: World Health Organization
