# Supplementary material for: Lessons from past pandemics: a systematic review of evidence-based, cost-effective interventions to suppress COVID-19
Source: Syst Rev. 2022 May 12;11:90. doi: 10.1186/s13643-022-01958-9 (PMC9096744; doi:10.1186/s13643-022-01958-9)
Supplement: Supplementary file 1 — Additional file 1. Result of the search. Description of 3-stage framework. [file 13643_2022_1958_MOESM1_ESM.docx]

**SUPPLEMENT—RESULT OF THE SEARCH**

| **Step** | **Searches** | **Results** |
| --- | --- | --- |
| 1 | pandemic control.mp. [mp=ti, ab, hw, tn, ot, dm, mf, dv, kw, fx, dq, nm, kf, ox, px, rx, ui, sy] | 108 |
| 2 | pandemic interventions.mp. [mp=ti, ab, hw, tn, ot, dm, mf, dv, kw, fx, dq, nm, kf, ox, px, rx, ui, sy] | 15 |
| 3 | non-pharmaceutical interventions.mp. [mp=ti, ab, hw, tn, ot, dm, mf, dv, kw, fx, dq, nm, kf, ox, px, rx, ui, sy] | 283 |
| 4 | outbreak control.mp. [mp=ti, ab, hw, tn, ot, dm, mf, dv, kw, fx, dq, nm, kf, ox, px, rx, ui, sy] | 1314 |
| 5 | epidemic control.mp. [mp=ti, ab, hw, tn, ot, dm, mf, dv, kw, fx, dq, nm, kf, ox, px, rx, ui, sy] | 981 |
| 6 | epidemic interventions.mp. [mp=ti, ab, hw, tn, ot, dm, mf, dv, kw, fx, dq, nm, kf, ox, px, rx, ui, sy] | 30 |
| 7 | outbreak interventions.mp. [mp=ti, ab, hw, tn, ot, dm, mf, dv, kw, fx, dq, nm, kf, ox, px, rx, ui, sy] | 35 |
| 8 | 1 or 2 or 3 or 4 or 5 or 6 or 7 | 2742 |
| 9 | remove duplicates from 8 | 1653 |

**SUPPLEMENT—DESCRIPTION OF 3-STAGE FRAMEWORK**


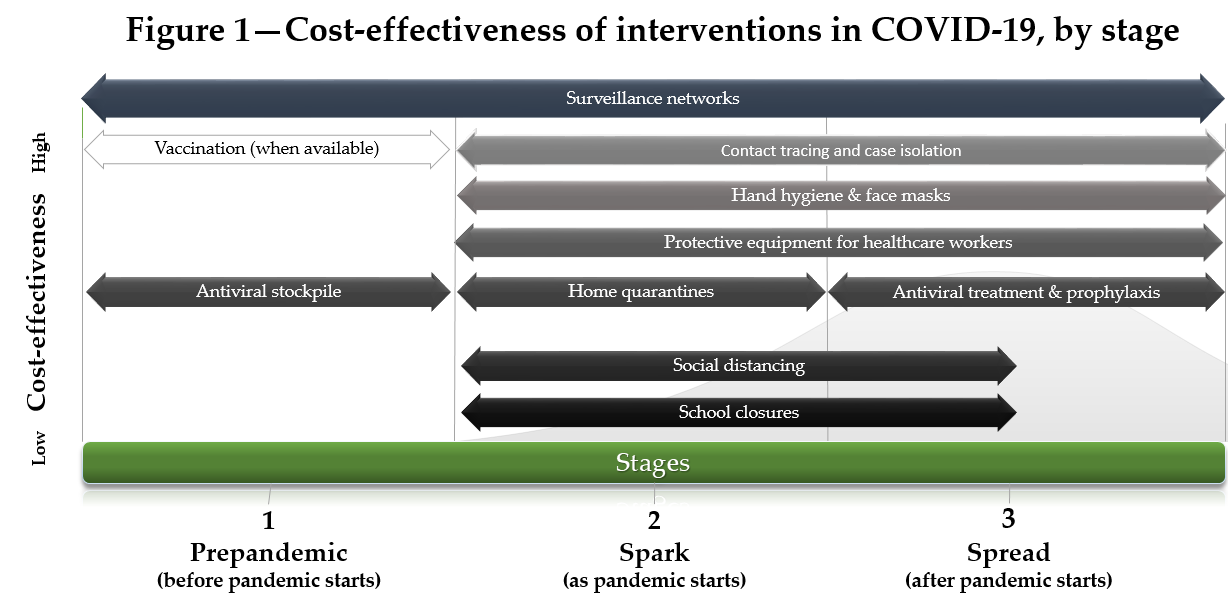


In Figure 1, we propose a 3-stage framework for pandemic control interventions. Interventions are shown from top (most cost-effective) to bottom (least cost-effective), according to the stages described by Madhav et al. (2017) as pre-pandemic, spark, and spread (shown from left to right). According to this framework, surveillance networks are highly cost-effective, should be established before the pandemic starts (stage 1), and maintained through stages 2 and 3. Vaccination, when available, should occur before the pandemic, or as early as possible. Antivirals can be stockpiled cost-effectively in high-income countries. As the pandemic starts (stage 2), early contact tracing and case isolation is the most cost-effective intervention. It may be sufficient to contain the outbreak. If the outbreak is not contained, hand hygiene, face masks, and protective equipment for healthcare workers are all highly cost-effective. If these measures are not sufficient, home quarantines, social distancing, and school closure are all effective, albeit increasingly costly measures. Assuming a 1 to 2% case fatality rate for COVID-19, these measures are likely to be cost-effective nonetheless, especially if implemented early. As COVID-19 spreads (stage 3), and especially past the peak, the costliest interventions can be replaced cost-effectively by a combination of interventions centered on swift contact tracing and case isolation. Once antivirals are available, they can also replace the costlier interventions cost-effectively.
