# Supplementary material for: Lessons from past pandemics: a systematic review of evidence-based, cost-effective interventions to suppress COVID-19
Source: Syst Rev. 2022 May 12;11:90. doi: 10.1186/s13643-022-01958-9 (PMC9096744; doi:10.1186/s13643-022-01958-9)
Supplement: Supplementary file 2 — Additional file 2. PRISMA Flow Diagram. [file 13643_2022_1958_MOESM2_ESM.doc]

**eFigure—PRISMA Flow Diagram**

**Screening**

**Included**

**Eligibility**

**Identification**

Records after duplicates removed
(n = 1,653)

Records identified through database searching
(n = 2,742)

Additional studies identified through other sources
(n = 23)

Records screened
(n = 622)

Records excluded
(n = 485)

Full-text articles assessed for eligibility
(n = 137)

Full-text articles excluded
(n = 98)

Studies included
(n = 62)
